# Influence of hydrocarbon oil structure on adjuvanticity and autoimmunity

**DOI:** 10.1038/s41598-017-15096-z

**Published:** 2017-11-08

**Authors:** Anthony C. Y. Yau, Erik Lönnblom, Jianghong Zhong, Rikard Holmdahl

**Affiliations:** 1 Division of Medical Inflammation Research, Department of Medical Biochemistry and Biophysics, Karolinska Institutet, SE-171 77 Stockholm, Sweden; 2 Present Address: Department of Immunology, Genetics and Pathology, Uppsala University, SE-751 85 Uppsala, Sweden

## Abstract

Mineral oils are extensively used in our daily life, in food, cosmetics, biomedicine, vaccines and in different industrial applications. However, exposure to these mineral oils has been associated with immune adjuvant effects and the development of autoimmune diseases. Here we investigate the structural impacts of the hydrocarbon oil molecules on their adjuvanticity and autoimmunity. First, we showed that hydrocarbon oil molecules with small atomic differences could result in experimental arthritis in DA rats differing in disease severity, incidence, weight change and serum levels of acute phase proteins. Injection of these hydrocarbon oils resulted in the activation, proliferation and elevated expression of Th1 and especially Th17 cytokines by the T cells, which correlate with the arthritogenicity of the T cells. Furthermore, the more arthritogenic hydrocarbon oils resulted in an increased production of autoantibodies against cartilage joint specific, triple-helical type II collagen epitopes. When injected together with ovalbumin, the more arthritogenic hydrocarbon oils resulted in an increased production of αβ T cell-dependent anti-ovalbumin antibodies. This study shows the arthritogenicity of hydrocarbon oils is associated with their adjuvant properties with implications to not only arthritis research but also other diseases and medical applications such as vaccines in which oil adjuvants are involved.

## Introduction

Mineral oils are often used in food, cosmetics, biomedicine and different industrial applications. However, such oils also have adjuvant properties and have been included in vaccines formulation to enhance immune responses. Importantly, intake of hydrocarbon oils, depending on the amount and route, can result in severe inflammatory reactions such as skin necrosis, loss of hand function, lipogranulomas in lung, lymph nodes and liver^1,2^. Exposure to mineral oils has been associated with an increased risk of developing rheumatoid arthritis (RA) and possibly lupus^3,4^, and intradermal administration of mineral oils can induce arthritis in susceptible rat strains, hereafter referred to as ‘adjuvant-induced arthritis’^5,6^. In other arthritis models, different antigens are injected together with the oil adjuvants, such as Freund’s incomplete adjuvant (IFA) and Freund’s complete adjuvant (CFA). These arthritis models include cartilage-restricted antigen-induced arthritis, which use type II collagen (CII), type XI collagen (CXI) or cartilage oligomeric matrix protein (COMP) as the antigen; and mycobacterial adjuvant-induced arthritis (Mbt-AIA), which induces disease by injection of heat-killed mycobacteria emulsified in IFA. These arthritis models mimic different aspects of RA and have been very useful for identifying arthritis-regulating loci and genes, many of which could not be detected in the past human genome-wide association studies due to various limitations^7,8^. Most arthritis loci regulate multiple arthritis models^9^, while some loci regulate only certain types of arthritis models^10–12^. A better understanding of these disease models may thus give invaluable information on the regulatory mechanisms of these disease genes.

For adjuvant-induced arthritis models, different immunostimulatory agents have been described to induce polyarthritis in arthritis-susceptible rat strains, such as DA. These agents include IFA, which is an undefined mixture of oil molecules (oil-induced arthritis, OIA), and also structurally defined hydrocarbon molecules such as pristane (pristane-induced arthritis, PIA), hexadecane (hexadecane-induced arthritis, HXIA) and squalene (squalene-induced arthritis, SIA)^5,6,11,13,14^. These show that non-specific stimulation of the immune system with oil adjuvants alone can elicit joint-specific inflammation. Similar to RA in humans, the susceptibility to these arthritis models are regulated by genes both within and outside the major histocompatibility complex (MHC)^10,11,13–16^ and are T cell dependent^17,18^. The exact pathogenic mechanism of these adjuvants remains unclear, although there have been different suggestions^19–23^.

In this study, we compared the elicited immune response at several time points after the injection of different oil adjuvants, including the more widely used ones such as pristane and squalene. With slight structural variation in these oil molecules, we could show that they differed significantly in not only the progression of the induced disease but already in the early phase with different extents of cell expansion, activation and proliferation and also expression of proinflammatory cytokines which correlates with the arthritogenicity of the adjuvant-primed CD4^+^ T cells. At the peak of arthritis, autoantibody response could be detected, with a stronger response developed in rats injected with the more arthritogenic adjuvants. In addition, we showed that these hydrocarbon adjuvants vary in their stimulatory effects on antigen-specific recall response and antibody production.

## Results

### Variation in arthritogenicity of different adjuvants

**Figure 1:**
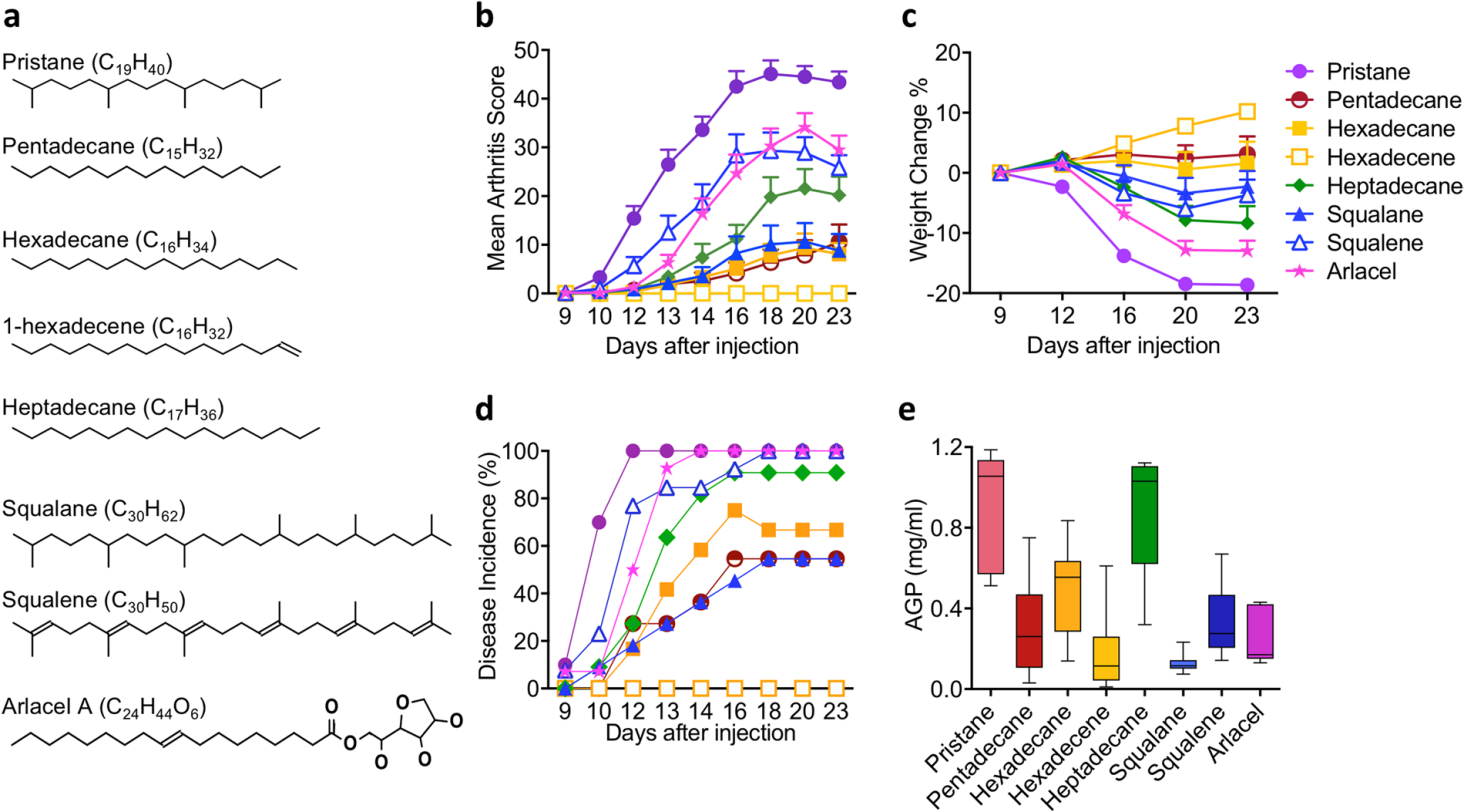
Development of arthritis in DA rats upon the injection of pristane (n = 10), pentadecane (n = 11), hexadecane (n = 12), hexadecene (n = 9), heptadecane (n = 11), squalane (n = 11), squalene (n = 13) and arlacel A (n = 14). (**a**) Structure of different oil adjuvants, (**b**) mean arthritis score ± SEM; (**c**) mean weight change percentage (±SEM) compared to day 9 after injection; (**d**) disease incidence (%) and (**e**) serum AGP level on day 23 after injection. Statistics were determined with the Mann-Whitney *U* test. *Denotes p < 0.05, **denotes p < 0.01, ***denotes p < 0.001.

We first evaluated the effect of different structures of oil adjuvants (Fig. 1a) on arthritis development in an arthritis-susceptible rat strain, DA. As shown in Fig. 1b,c, pristane is the most arthritogenic among all the tested adjuvants, resulting in the highest mean arthritis score and greatest weight reduction. Arthritis starts on day 9 and reaches 100% incidence on day 12 (Fig. 1d). Pentadecane, structurally identical to pristane except the absence of four methyl groups on its 15-carbon backbone, induces significantly milder arthritis with lower mean arthritis score, reduced weight reduction and lower disease incidence (Fig. 1b–d). Hexadecane, which is slightly longer than pentadecane with a 16-carbon backbone, induces mild arthritis, similar to pentadecane. Nevertheless, heptadecane with 17-carbon backbone induces more severe arthritis than pentadecane and hexadecane (Fig. 1b–d). The presence of a carbon-carbon double bond in hexadecene, however, renders the hydrocarbon non-arthritogenic. In contrast, the presence of a carbon-carbon double bonds in squalene enhance arthritis since the unsaturated squalene developed more severe arthritis than the saturated squalane (Fig. 1b–d). Arlacel A (also named mannide monooleate), an emulsifying agent in IFA, induces severe arthritis as well (Fig. 1b–d).

We next determined the level of an acute phase protein in the serum, α_1_-acid glycoprotein (AGP). In general, the level of AGP correlates with the arthritogenicity of the adjuvants (Table 1). For example, the injection of the most arthritogenic adjuvants, such as pristane, resulted in a higher level of serum AGP, while the non-arthritogenic adjuvants, such as hexadecene, resulted in a lower level of serum AGP. However, this was not a strict correlation. For instance, injection of heptadecane resulted in nearly the highest level of serum AGP, even heptadecane did not induce the most severe form of arthritis. Injection of squalene and Arlacel A induced severe arthritis but resulted in rather low level of serum AGP compared to most other adjuvants (Fig. 1e).

**Table 1:** Autoantibody and AGP production and their correlations with arthritis development.

Epitope	Autoantibody response^1^		Spearman’s rank correlation coefficient^2^			
	Pristane^1^	IFA^1^	Max Score	Sample Day Score	Sum Score	Weight
CII_263–270_	10.32	5.20	0.2815**	0.3106***	0.2899***	−0.2657**
C1	8.70	3.32	0.4252****	0.4328****	0.4541****	−0.4356****
E10	5.53	3.89	0.2646**	0.2844**	0.2863***	−0.2971***
J1	5.20	2.94	0.3830****	0.4172****	0.3964****	−0.4040****
F4	4.01	2.50	0.3394****	0.3461****	0.3436****	−0.3652****
U1	3.48	2.37	0.3940****	0.3935****	0.4000****	−0.4170****
E17	3.01	2.22	0.2788**	0.3124***	0.3075***	−0.3291***
Filaggrin	2.17	2.23	0.1682	0.1693	0.1683	−0.2660**
Vim_1–20_	1.83	1.82	0.0947	0.0935	0.1112	−0.2093
Vim_58–77_	1.83	1.82	0.1752*	0.1794*	0.1752*	−0.2295**
Fib_501–517_	1.77	2.05	0.0388	0.0555	0.0283	−0.1369
α-enolase	1.65	1.74	0.1074	0.0905	0.1232	−0.2115*
Fib_60–78_	1.64	1.74	0.1498	0.1498	0.1534	−0.2459**
Fib_617–635_	1.49	1.80	0.2549**	0.2518**	0.2872***	−0.3616****
AGP^3^			0.4557****	0.5643****	0.4770****	−0.5210****

^1^The autoantibody response was measured as MFI_adjuvant_/MFI_naive_, where MFI_adjuvant_ and MFI_naive_ are the MFI of the corresponding epitopes in the serum harvested from rats injected with adjuvant and from naive rats respectively.

^2^Only adjuvant induced arthritis samples were included (n = 131). CIA and naive samples are not included in the correlation analysis. *Denotes p < 0.05, **denotes p < 0.01, ***denotes p < 0.001, ****denotes p < 0.0001.

^3^Data obtained from AGP ELISA kit.

### Variation in arthritogenicity of T cells primed with different adjuvants

The variation in disease onset and progression suggested different arthritogenic T cell activation by different oil adjuvants. In an adoptive cell transfer experiment, we previously showed that T cells from DA rats required a minimum of 5 days of pristane-priming *in vivo* to transfer arthritis and 8 days for the T cells to become fully arthritogenic^24^. Based on Fig. 1, we selected four oil adjuvants which varies in arthritogenicity, namely pristane, hexadecane, squalane and squalene, and studied the arthritogenic capacity of T cells that were previously primed *in vivo* with these adjuvants. Draining lymph node cells were collected 5 and 8 days post injection (p.i.) and stimulated with concanavalin A *in vitro*, before being adoptively transferred into naive recipient rats. Macroscopic arthritis score, weight change and disease incidence was assessed in these recipient rats.

**Figure 2:**
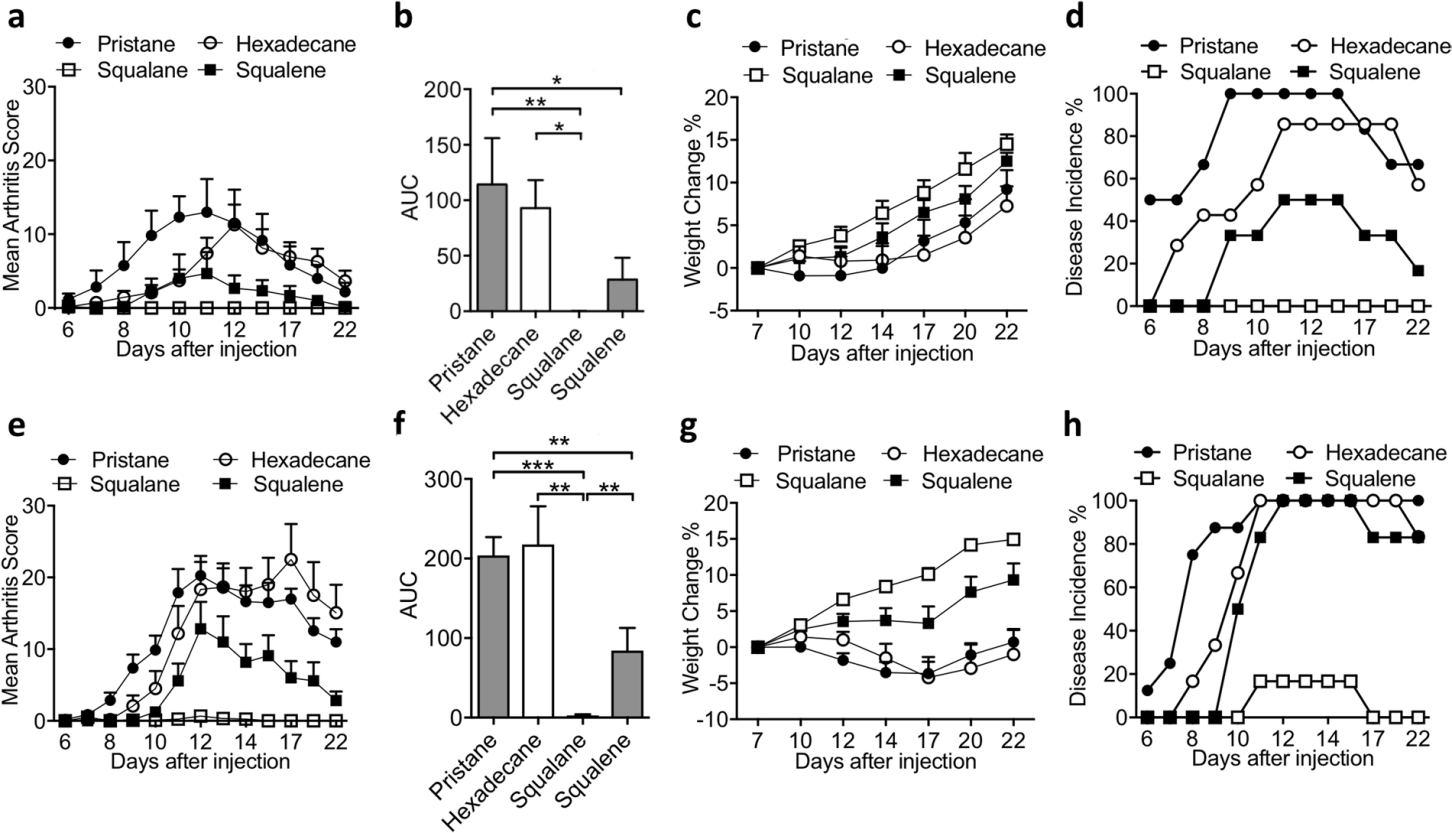
Variation in the arthritogenicity of T cells *in vivo* primed with pristane, hexadecane, squalane and squalene for (**a**–**d**) 5 days and (**e**–**h**) 8 days, n = 5–8 per group. (**a**,**e**) mean arthritis score ± SEM; (**b**,**f**) area under the arthritis score curve (±SEM) of Fig. 2a and e respectively; (**c**,**g**) mean weight change percentage (±SEM) compared to day 7 after injection; (**d**,**h**) disease incidence (%). Statistics were determined with the Mann-Whitney *U* test. *denotes p < 0.05, **denotes p < 0.01, ***denotes p < 0.001.

As expected, T cells harvested 8 days p.i. transferred more severe arthritis with higher disease incidence and a longer disease course than T cells harvested 5 days p.i. for all tested oil adjuvants (Fig. 2). This reflects the increase in arthritogenic capacity of the adjuvant-primed cells with disease progression. In both day 5 (Fig. 2a–d) and day 8 (Fig. 2e–h) transfer, pristane-primed T cells are the most arthritogenic, resulting in the recipient rats developing the most severe arthritis with the most weight reduction, the earliest day of disease onset and 100% disease incidence. Hexadecane-primed T cells are more or less as arthritogenic as the pristane-primed T cells. Squalene-primed T cells are less arthritogenic than the pristane-primed and hexadecane-primed T cells, resulting in less severe arthritis with later disease onset in the recipient rats. Squalane-primed T cells are not arthritogenic on day 5 p.i., and only 1 out of 6 rats injected with T cells *in vivo* primed with squalane for 8 days developed arthritis, which was also very mild.

### Arthritogenic adjuvant administration results immune cell expansion and activation and proliferation of CD4^+^ T cells

**Figure 3:**
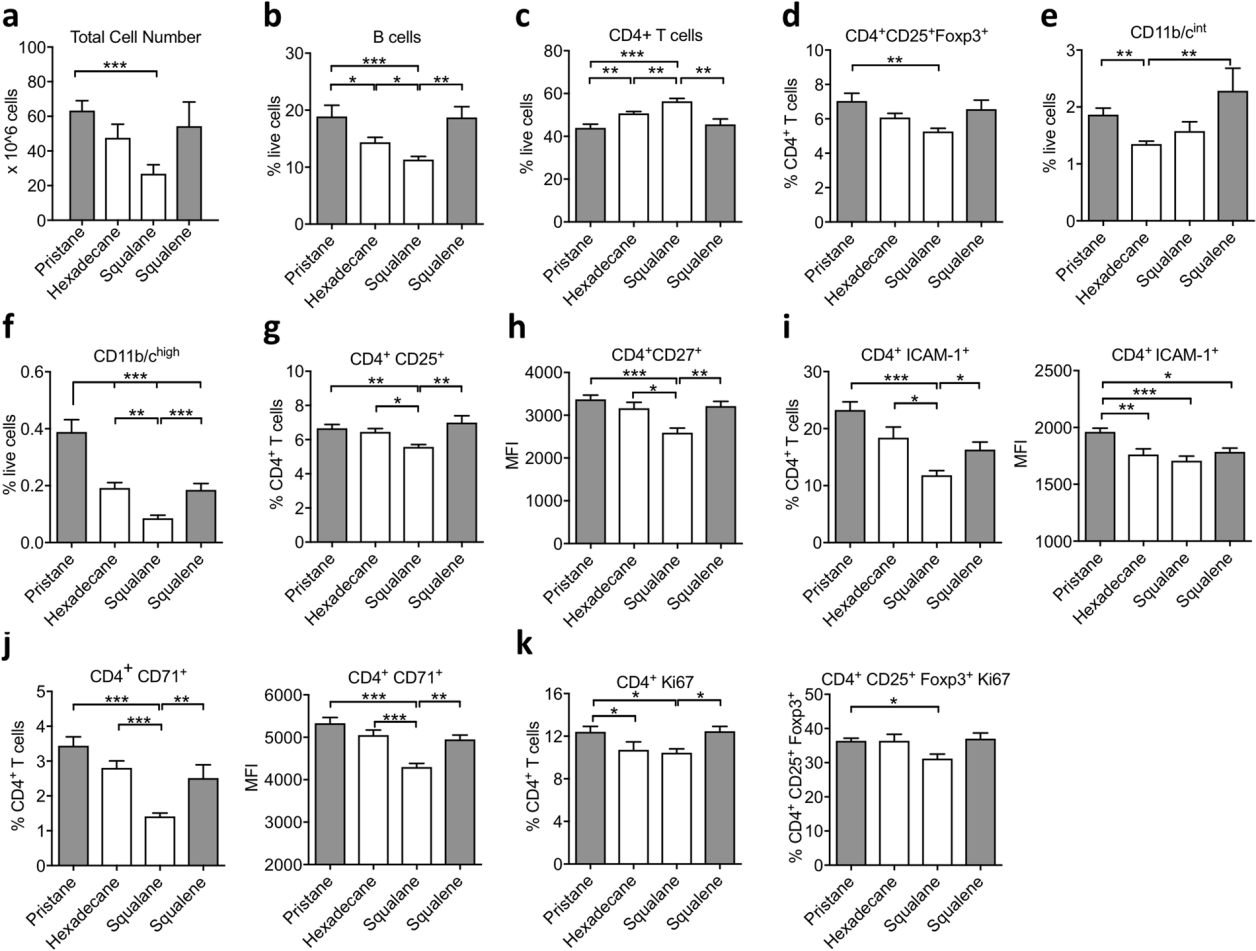
Immune cell frequency and expression of activation and proliferation markers on inguinal lymph nodes from DA rats primed with pristane, hexadecane, squalane and squalene for 5 days (n = 8 per group). (**a**) Total cell number, (**b**) B cells (%), (**c**) CD4 + T cells (%), (**d**) CD4^+^CD25^+^Foxp3^+ ^T cells (%), (**e**) CD11b/c^int^ (%), (**f**) CD11b/c^high^ (%). (**g**–**k**) CD4^+^ T cell expression of (**g**) CD25^+^ (%), (**h**) CD27^+^ (MFI), (**i**) ICAM-1^+^(%, MFI), (**j**) CD71^+^ (%, MFI), (**k**) Ki67^+^ (%) on CD4^+^ T cells and Ki67^+^ (%) on CD4^+^CD25^+^Foxp3^+^ T cells. Data are shown as mean ± SEM. Statistics were determined with the Mann-Whitney *U* test. *Denotes p < 0.05, **denotes p < 0.01, ***denotes p < 0.001.

We next performed flow cytometry to investigate the effect of oil adjuvants on different immune cell populations, in particular T cells, in the lymph nodes on day 5 p.i. (Fig. 3) and day 8 p.i. (Supplementary Fig. 2).

We first found that injection of different adjuvants resulted in different extent of cell expansion. The total number of lymph node cells in pristane-primed rats increased to 60 million on day 5 p.i. and 110 million cells on day 8 p.i., which is much greater than the rats injected with squalane that have only 30 million cells on day 5 p.i. and 50 million cells on day 8 p.i. (Fig. 3a, Supplementary Fig. 2). There were also more rapid expansion in the number of B cells, CD11b/c^int^ cells and CD11b/c^high^ cells (which are mainly monocytes and neutrophils respectively) in pristane- and squalene-primed rats on day 5 p.i. (Fig. 3b,e,f). Unexpectedly, the frequency of the CD4^+^ T cells are lower in pristane- and squalene-primed rats than that of squalane-primed rats (Fig. 3c), although the absolute number of CD4^+^ T cells remain higher in pristane- and squalene-primed rats (data not shown).

While we did not detect difference in the activation and MHC class II expression on the classical antigen presenting cells (data not shown), we found that injection of different adjuvants resulted in different degree of activation and proliferation of T cells. Pristane-primed CD4^+^ T cells were the most activated and proliferative, while squalane-primed CD4^+^ T cells were the least activated and proliferative, as determined by a number of activation and proliferation markers on day 5 after injection. Pristane-primed CD4^+^ T cell had the highest expression of CD25, CD27, CD71, ICAM-1 and Ki67; whereas squalane-primed CD4^+^ T cells exhibited the lowest expression of these markers (Fig. 3g–k). Squalene-primed CD4^+^ T cells are also significantly more activated and proliferative than the squalane-primed CD4^+^ T cells. Compared with squalane-primed CD4^+^ T cells, pristane-primed and squalene-primed CD4^+^ T cells comprised a higher frequency of regulatory T cells (CD3^+ ^CD4^+^ CD25^+^ Foxp3^+^) (Fig. 3d) which were also more proliferative (Fig. 3k). This could be necessary to counteract the increased expansion of autoreactive T cells in rats injected with pristane.

Overall, the pattern of activation and proliferation of the CD4^+^ T cells on day 5 p.i. was also similarly found on day 8 p.i (Supplementary Fig. 2). Comparing the result of the adoptive cell transfer experiment (Fig. 2) and flow cytometry analysis of the lymph node cells (Fig. 3), the activation and proliferation of T cells largely correlate to the arthritogenicity of the CD4^+^ T cells, i.e. more activated and proliferative T cells transfer more severe arthritis.

### Th17 cytokine gene expression correlates with the arthritogenicity of CD4^+^ T cells

**Figure 4:**
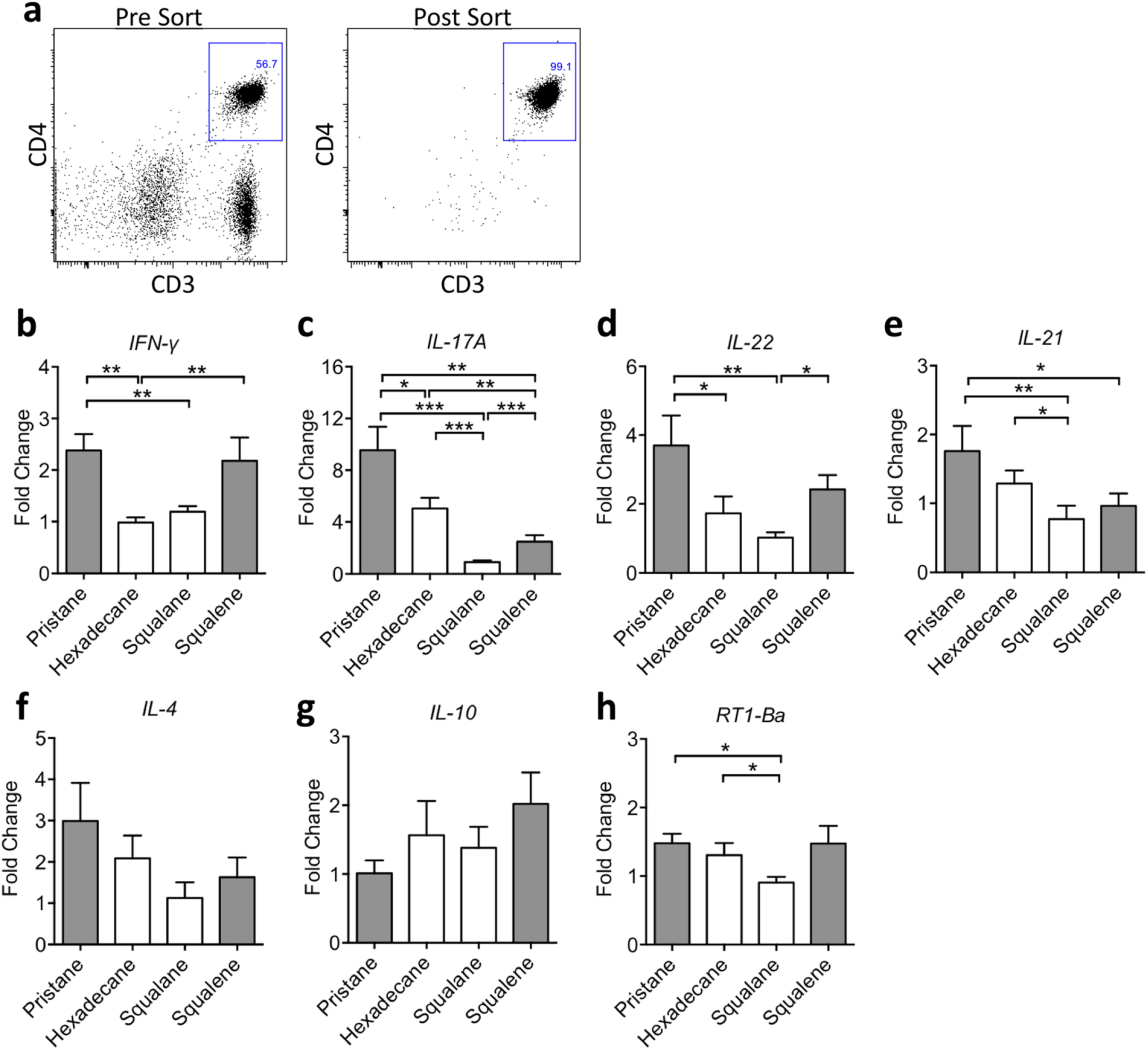
Cytokine and MHC class II expression of isolated CD4^+^ T cells on day 5 after injection of different oil adjuvants. (**a**) Representative flow cytometry plot showing the isolation of CD4^+^ T cells before and after sorting. (**b**–**h**) Transcript level as determined by quantitative real-time PCR on isolated CD4 + T cells primed with adjuvants pristane, hexadecane, squalane and squalene for 5 days (n = 8 per group): (**b**) *IFN-γ*, (**c**) *IL-17A*, (**d**) *IL-22*, (**e**) *IL-21*, (**f**) *IL-4*, (**g**) *IL-10*, (**h**) *RT1-Ba*. Data are shown as mean ± SEM. Statistics were determined with the Mann-Whitney *U* test. *Denotes p < 0.05, **denotes p < 0.01, ***denotes p < 0.001.

Having analysed different immune cell populations and shown that different adjuvant-primed CD4^+^ T cells differed in their activation and proliferation on day 5 p.i. (Fig. 3g–k), we determined mRNA levels of cytokines and MHC class II in purified CD4^+^ T cells also on day 5 p.i. (Fig. 4a). We found that the more arthritogenic oil adjuvants pristane and squalene induced significantly higher expression of the proinflammatory cytokine *IFN-γ* than the less arthritogenic oil adjuvants such as hexadecane or squalane (Fig. 4b). Further, the *IL-17A* gene expression pattern (Fig. 4c) correlates very well with the arthritogenicity of CD4^+^ T cells (Fig. 2b), with similar gene expression pattern shown for other Th17 cytokines *IL-22* and *IL-21* (Fig. 4d,e). Pristane-primed, which resulted in the most severe arthritis in the recipient rats, measured with AUC (area under the curve), exhibited the highest *IL-17A* gene expression (Figs 2b and 4c). Hexadecane-primed T cells, with the second highest AUC, has the second highest *IL-17A* gene expression (Figs 2b and 4c). This is followed by squalene-primed T cells, and squalane-primed T cells with the lowest AUC inducing the lowest *IL-17A* gene expression (Figs 2b and 4c). There is however no difference in the expression of Th2 cytokines *IL-4* and *IL-10* (Fig. 4f,g). Among all oil adjuvants, squalane induces significantly lowest MHC class II expression (Fig. 4h), which has been reported in activated rat T cells^25^. When stimulated with anti-CD3/anti-CD28, squalane-primed lymph node cells produced significantly less IFN-γ and IL-17 compared with other adjuvants (Supplementary Fig. 1). All these data point to the importance of Th1 and especially Th17 lineage in rats in the development of arthritogenic response in adjuvant-induced arthritis models.

### Adjuvant induction of autoantibody response against type II collagen and other antigens

The discovery that a large proportion of patients with RA develop autoantibody response has led to extensive research into the use of such autoantibody response as diagnostic and prognostic biomarkers in both humans and rodents^26^. Whereas it is known that adjuvant-induced arthritis models such as PIA is driven by autoreactive T cells, it has been difficult to identify the autoantigens involved in the pathogenesis of arthritis with only very few such autoantigens that have been identified^15,20^. We recently developed a sensitive assay for detecting antibody response (measured as mean florescence intensity, MFI) against a number of autoantigen-derived peptides, including epitopes in triple-helical CII, α-enolase, filaggrin, vimentin, and fibrinogen. Using this assay, we detected autoantibody response in rats injected with different hydrocarbon oil adjuvants, and we used serum from rats with CIA or OIA, and also from naive rats as a control.

**Figure 5:**
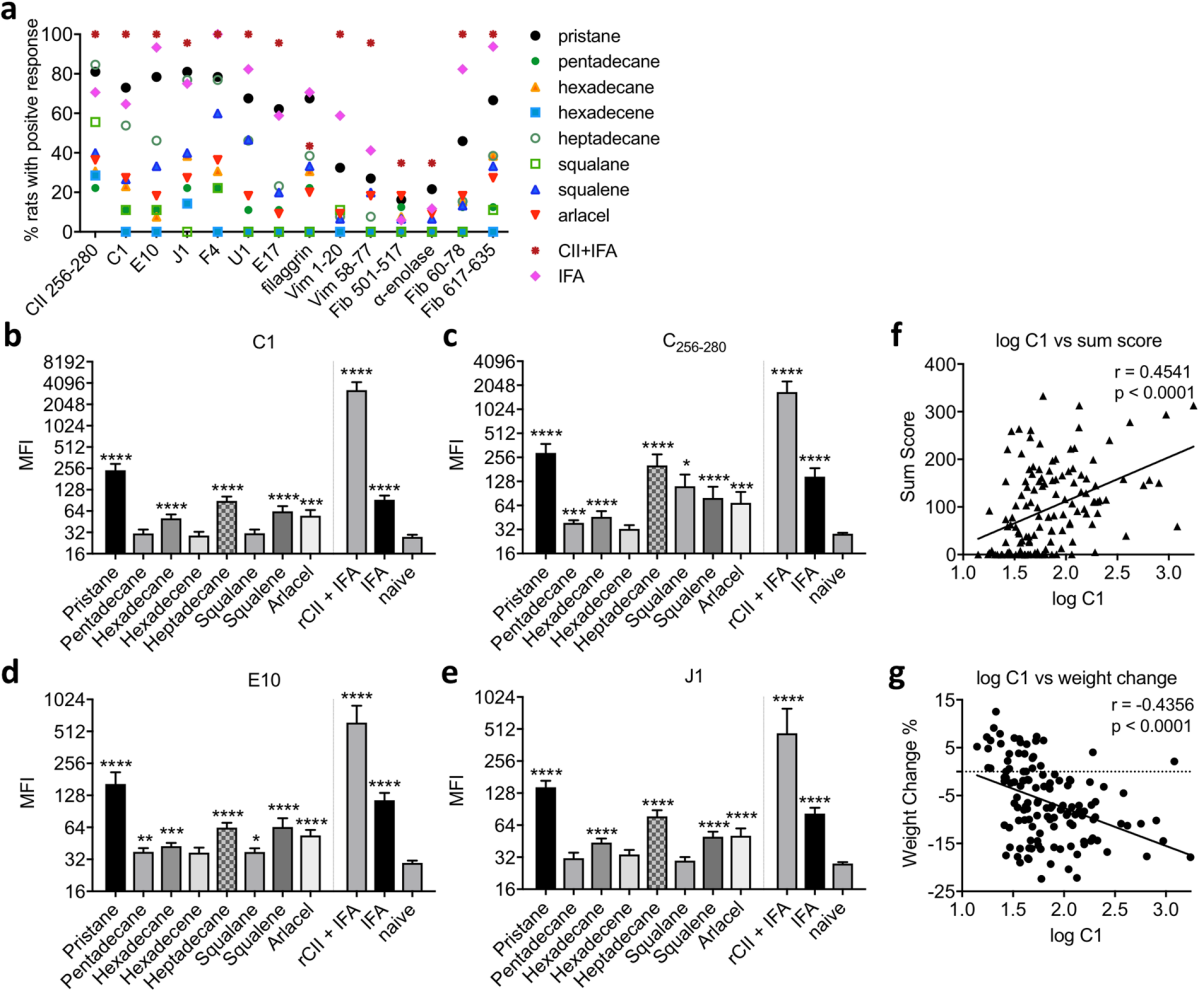
Antoantibody response in DA rats on day 23 after adjuvant administration. (**a**) Percentage of rats injected with adjuvants that developed autoantibody response against CII_256–280_, C1, E10, J1, F4, U1, E17, filaggrin, Vim_1–20_, Vim_58–77_, Fib_501–517_, α-enolase, Fib_60–78_ and Fib_617–635_; (**b**–**e**) mean florescence intensity (MFI) measurement of autoantibody response against different collagen epitopes (**b**) CII_256–280_, (**c**) C1, (**d**) E10, (**e**) J1; (**f**) correlation plot of sum arthritis score versus log (anti-C1 antibody); and (**g**) correlation plot of weight change (%) versus log (anti-C1 antibody). (**b**–**f**) Data are shown as mean ± SEM. Statistics were determined with the Mann-Whitney *U* test. *Denotes p < 0.05, **denotes p < 0.01, ***denotes p < 0.001. (**g**,**h**) Spearman’s correlation coefficient r and the corresponding p value is shown in each figure.

In general, a larger proportion of rats injected with the more arthritogenic oil adjuvants such as pristane and heptadecane, developed autoantibody response than rats injected with the non-arthritogenic or weakly arthritogenic adjuvants such as hexadecene and pentadecane (Fig. 5a). Furthermore, as measured in MFI_adjuvant_/MFI_naive_ ratio, the autoantibody responses against cartilage joint specific triple-helical CII epitopes (CII_256–280_, C1, E10, J1, F4, U1 and E17) were stronger than those against other antigens such as α-enolase, filaggrin, vimentin and fibrinogen (Table 1) and more rats developed autoantibody response against CII than other antigens (Fig. 5a–e).

As shown in Table 1, the four CII epitopes with the strongest autoantibody responses are CII_256–280_, C1, E10, and J1; and the size of the antibody response against each of these epitopes for each oil adjuvant, as measured in MFI, is illustrated in Fig. 5b–e. We investigated if there is any correlation between autoantibody response and arthritis severity in the rats injected with hydrocarbon oil adjuvants using criteria including maximum arthritis score, sampling day arthritis score, arthritis sum score and weight change (Table 1). Among all the assessed autoantibody responses, antibodies against C1 showed the strongest correlation to the development of arthritis (arthritis sum score: r = 0.45, p < 0.0001) (Fig. 5g,h, Table 1). The strength of correlation is similar to the known serum marker AGP (arthritis sum score: r = 0.48, p < 0.0001) (Table 1). Antibodies against C1 have been shown to be pathogenic and are used to induce an acute form of arthritis in the collagen antibody-induced arthritis (CAIA) model^27^. In this study, we showed that antibodies against C1 could also be detected in adjuvant-induced arthritis, despite at a lower level than CIA, and that C1 antibodies positively correlated with the severity of arthritis.

One of the diagnostic criteria in RA is the level of anti-citrullinated protein antibodies (ACPA) in serum^28^, although most studies so far have suggested that there are little or no ACPAs in rodents. We assessed antibody response against different citrullinated peptides identified in RA patients, including triple-helical CII, α-enolase, filaggrin, vimentin, and fibrinogen^26,29^. We did not detect any significant increase in the antibody response against nearly all of citrullinated epitopes in all 37 PIA and 21 CIA serum samples (Supplementary Figs 3 and 4).

### The effect of adjuvants on antigen-specific recall response and antibody production

**Figure 6:**
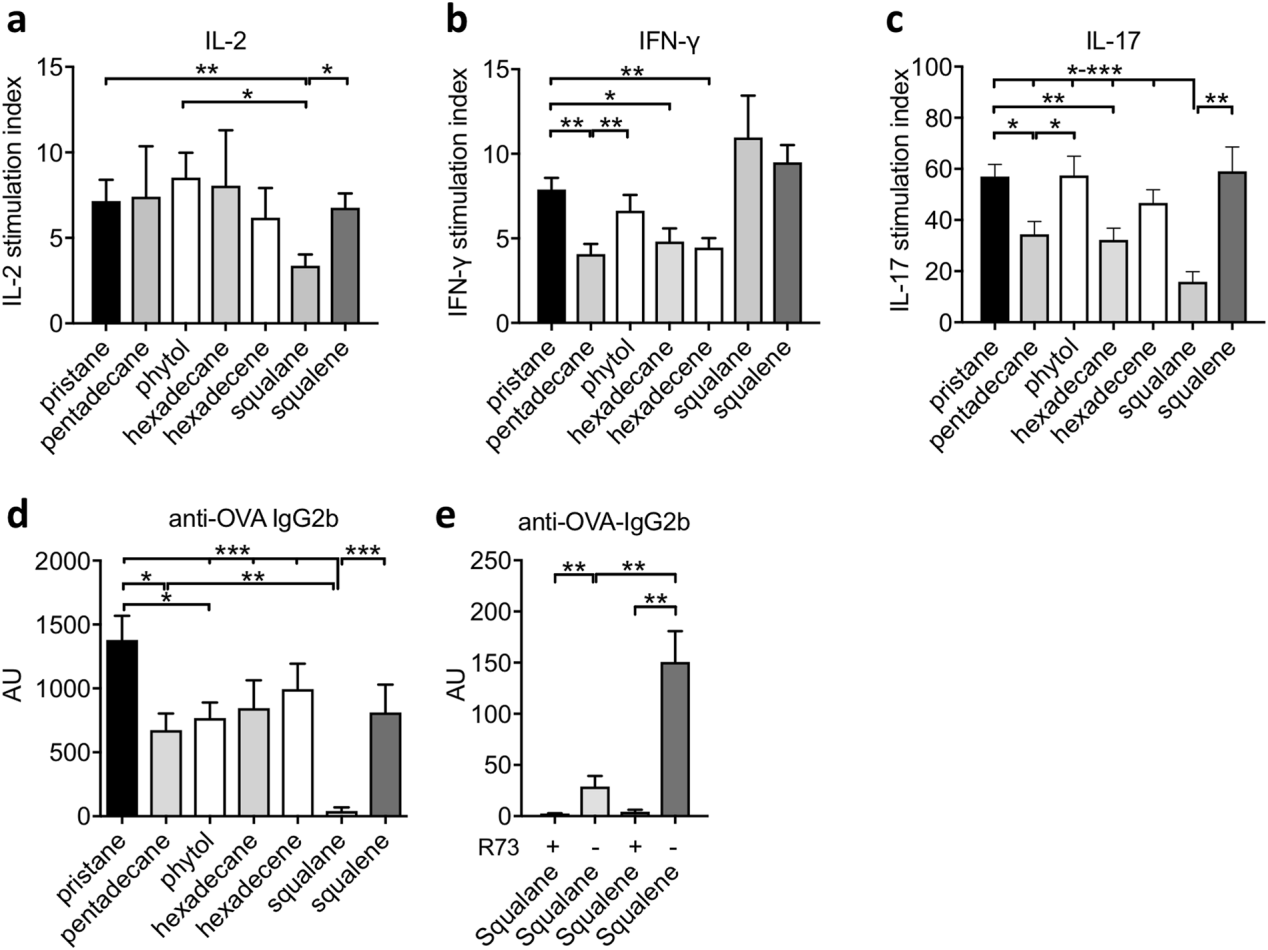
Effect of adjuvants on ovalbumin-specific recall response and antibody production. (**a**–**d**) DA rats were injected with ovalbumin together with pristane, pentadecane, phytol, hexadecane, heptadecane, squalane and squalene (n = 8 per group). Draining lymph nodes were harvested 10 days after immunization and restimulated *in vitro* with ovalbumin. Level of cytokines in the supernatant was determined as a measure of T cell recall response. (**a**) IL-2 was measured after 20 hours, and (**b**) IFN-γ and (**c**) IL-17 were measured after 88 hours of restimulation with ovalbumin. The stimulation index was determined by dividing the level of cytokines produced by the cells cultured in medium with ovalbumin, by the level of cytokines produced by the cells that were cultured in medium without ovalbumin. (**d**) Levels of ovalbumin-specific antibodies IgG2b in the serum was determined by ELISA and shown as arbitrary unit (AU). (**e**) Levels of ovalbumin-specific antibodies IgG2b in the serum in rats injected with ovalbumin together with squalane and squalane, with and without αβ T cell depletion (n = 5–6 per group). Data are shown as mean ± SEM. Statistics were determined with the Mann-Whitney *U* test. *Denotes p < 0.05, **denotes p < 0.01, ***denotes p < 0.001.

We investigated the effect of oil adjuvants on antigen-specific recall response by injecting rats with ovalbumin (as the antigen) and different oil adjuvants. Ten days after immunization, we restimulated the draining lymph node cells *in vitro* with ovalbumin, and determined the level of various cytokines in the supernatant as a measure of T cell recall response, including IL-2 after 20 hours and interferon-gamma (IFN-γ) and IL-17 after 88 hours. We found that the choice of adjuvants did have a profound effect on the ovalbumin-specific recall response (Fig. 6a–c), in particular, in the production of IFN-γ and IL-17. The use of most arthritogenic adjuvants such as pristane and squalene *in vivo* had the strongest stimulatory effect on the recall response, compared with the use of weakly arthritogenic adjuvants, such as pentadecane and hexadecane (Fig. 6b,c). Three of the adjuvants however did not strictly follow this trend. The non-arthritogenic adjuvant hexadecene resulted in high production of IL-17 (Fig. 6c); and the weakly arthritogenic adjuvant squalane resulted in the highest production of IFN-γ (Fig. 6b). In addition, we included a second non-arthritogenic adjuvant, phytol (Supplementary Fig. 5)^30^, and showed that phytol led to a strong stimulatory effect on the recall response as measured by IFN-γ and IL-17 (Fig. 6b,c).

We also assessed the serum level of ovalbumin-specific antibody response. While the oil adjuvants used have no effect on the level of OVA-specific IgG1 (data not shown), the choice of adjuvants has a pronounced effect on the level of OVA-specific IgG2b (Fig. 6d), an indication of Th1 response^31^. Using pristane as an adjuvant resulted in the highest titer of OVA-specific IgG2b. Other C_15–17_ oil adjuvants and squalene resulted in a lower titer; whereas squalane resulted in the lowest titer of OVA-IgG2b. We further evaluated the importance of αβ T cells on antibody response by *in vivo* depletion of αβ T cells. We showed that αβ T cell depletion resulted in almost complete abrogation of OVA-specific antibody response in both groups of rats injected with ovalbumin together with squalane or squalene (Fig. 6e), thus confirming the importance of the αβ T cells in the adjuvant regulation of antigen-specific antibody production.

## Discussion

In this study, we assess the structural impacts of the hydrocarbon oil molecules on their adjuvanticity and autoimmunity. We showed that atomic differences in the hydrocarbon oil adjuvants could result in dramatic differences in adjuvanticity and autoimmunity, including differences in arthritis induction, acute phase response, immune cell frequency, T cell activation, proliferation and cytokine expression, autoantibody production, and antigen-specific recall response and antibody production. Combining both gene expression and flow cytometry analysis, together with adoptive disease transfer of T cells on day 5 and day 8 after adjuvant administration, we showed the choice of oil adjuvants can have pronounced impact on immune cell responses which resulted in different disease outcomes and the arthritogenicity of hydrocarbon oils is associated with their adjuvant properties.

The fact that arthritis can be induced by adjuvants in the absence of other exogenous antigens makes adjuvant-induced arthritis an invaluable model to study the induction of T cell mediated autoimmunity. Adjuvant-induced arthritis show many similarities to human RA in being chronic (shown for pristane^6,17^ and squalene^14^), regulated by T cells and by being associated with the similar genetic loci, both MHC and non-MHC genes^5,7,16,32,33^. Importantly, the most strongly associated non-MHC gene *Ncf1*, which leads to increased arthritis severity with lower ROS response in the DA rat strain, is a genetic association that is clearly present also in human RA and systemic lupus erythematosus^32,34,35^. Adjuvants are normally present in our body. Pristane originates from exogenous sources. It is present in chlorophyll and is enriched in eukaryotic organisms, and are digested by both rats and humans. Squalene is the biochemical precursor of cholesterol and other steroids, present in sebum in animals but can also lead to chronic arthritis^14^. The challenge is to identify the pathogenic mechanism which these endogenous and ‘harmless’ adjuvants lead to inflammation. By studying structurally similar but pathogenically very different hydrocarbon adjuvants, we have the possibility to investigate the elements that are important in leading to joint inflammation. It is possible that whether a particular adjuvant can penetrate and cross phospholipid bilayer determines its arthritogenicity. Shorter hydrocarbon molecules (for example, shorter than C_15_ backbone)^23^ or hydrocarbons of certain shapes (for example, due to the presence or absence of carbon-carbon double bond) are not soluble in cell membranes and are thus less arthritogenic or non-arthritogenic. One of the most arthritogenic adjuvants, pristane, has been shown to interact with phospholipid bilayers and be incorporated into the plasma membrane^19,36^. Other known effects of pristane include promoting tumour growth^37^, activating the signal transducing protein kinase C important in cell growth and differentiation^38^ and also inducing cell death^21^.

Within 1 hour after injection, the injected adjuvants spread rapidly and reached different organs with high selectivity for lymph nodes and rather few oil droplets were distributed to joints^39^. At the lymph nodes, oil adjuvants lead to both local and systemic reaction including hyperplasia and an increased level of acute phase protein such as α_1_-acid glycoprotein (AGP) in the blood^14,17^. Here we clearly showed that the more arthritogenic adjuvants such as pristane induce stronger hyperplasia in the lymph node with the expansion of various cell types (as early as five days after adjuvant administration) accompanied by a stronger AGP response. Both PIA and OIA are known to be αβ T cell dependent and can be adoptive transferred by CD4^+^ T cells^18,40,41^. We showed that in addition to pristane-primed T cells, T cells *in vivo* primed with other adjuvants such as hexadecane and squalene can also transfer arthritis. The result of the adoptive cell transfer experiments however are not entirely as we anticipated. As expected, pristane-primed T cells are the most arthritogenic transferring the most severe form of arthritis, which is not surprising since we showed that pristane is the most arthritogenic adjuvants among all the adjuvants assessed in this study. What is unexpected is that, whereas we showed that hexadecane is a milder arthritogenic adjuvant compared with squalene (Fig. 1), hexadecane-primed T cells are more arthritogenic transferring more severe arthritis than squalene-primed T cells (Fig. 2). Furthermore, whereas hexadecane and squalane induces similarly mild arthritis (Fig. 1), hexadecane-primed T cells are fully arthritogenic (disease incidence 86% and 100% in day 5 and day 8 transfer respectively) but squalane-primed T cells are not (disease incidence 0% and 17% in day 5 and day 8 transfer respectively) (Fig. 2). All these together seem to indicate that T cells *in vivo* primed with pristane and hexadecane (which are smaller in molecular size) reach their full arthritogenic capacity at an earlier time point than the T cells *in vivo* primed with squalane and squalene (which are larger in molecular size). Exactly how structural differences in these adjuvant molecules result in different pattern of arthritis induction and arthritogenicity of CD4^+^ T cells require further investigation. Our analysis, however, showed that the most arthritogenic adjuvant-primed αβTCR^+^ CD4^+^ cells are the most activated and proliferative with the highest expression of Th1 and Th17 cytokines.

Another important field of adjuvant applications are their use in vaccines, where the use of vaccine adjuvants has also been associated with the elevated production of cytokines such as IFN-γ and IL-17^42^. Adjuvants are added into a vaccine to improve its immunogenicity. The challenge for developing vaccine is therefore to maximise the immunogenicity and to minimize vaccine reactogenicity and the risk of developing any ‘adjuvant-induced’ autoimmune or chronic degenerative disorders, such as narcolepsy^43–45^ or Gulf War syndrome^46^. It is known that oil adjuvants induce tissue damage and cell death at the injection site leading to the production of damage-associated-molecular patterns (DAMPs) and inflammasome activation. Here we demonstrated that different adjuvants exert different extent of boosting effect on antigen-specific recall response and antibody production. We showed the importance of αβ T cells on the immune boosting effect of adjuvants, since αβ T cell depletion almost completely abrogated any antigen-specific antibody production. The fact that adjuvants induce arthritis only in genetically susceptible strains^7,17^ and autoimmune disorders occurring only in genetically susceptible individuals^47^ shows adjuvant induction of autoimmune diseases is partly determined by genetic factors. In future, it will be beneficial to investigate if these genetic factors, some of which have been identified by animal studies in our group^7,11^, also determine whether particular individuals with certain genetic profiles carry a higher risk of developing any adverse reactions upon adjuvant-based vaccination.

Using the Luminex technology, we detected a stronger autoantibody response in rats injected with more arthritogenic adjuvants; and a stronger autoantibody response against triple helical CII peptides than other antigens. In addition, we found that the antibody responses against several CII epitopes, such as C1, significantly correlated to the development of arthritis. The pathogenic roles of antibodies binding to CII epitopes, such as C1, J1 and U1, have been studied and shown for collagen-induced arthritis^27,48–50^, but not for adjuvant-induced arthritis. These CII antibodies are not likely to be involved in arthritis induction since adjuvant-induced arthritis does not depend on B cells or antibody production^40,51^. It remains to be determined whether these autoantibodies play any pathogenic roles or are simply a consequence of the disease development in the adjuvant-induced arthritis models.

This study not only provides novel information on the effects of different hydrocarbon oil adjuvants but is also an example how we can utilize different animal disease models and experimental techniques to characterize different aspects of adjuvant actions in not only arthritis but also other applications which involve the use of oil adjuvants. In this investigation, we showed the effect of slight structural variation in the oil adjuvants can have on both adjuvanticity and autoimmunity. Future research on both cellular and molecular mechanisms underlying adjuvant action potentially help us define the mechanisms critical in driving autoimmune response and also a better understanding of the adjuvants used for vaccines.

## Methods

### Animals

Inbred DA/Ztm rats were obtained from the Zentralinstitut für Versuchstierzucht (Hannover, Germany) and DA/OlaHsd from Harlan Europe (Netherlands). Rats were maintained by sister-brother mating in a barrier facility at Scheele Laboratory, Karolinska Institutet, and were specific pathogen free according to the Federation of European Animal Laboratory Science Associations guidelines. All animals were housed in a climate-controlled environment with 14 h light/10 h dark cycles, in individually ventilated microisolator-cages (Allentown) containing wood shavings (Tapvei), and fed standard rodent chow (R70, Lantmännen) and had free access to water. 8–12 weeks old, age- and sex-matched rats were used in all experiments.

### Disease Induction and Evaluation

Arthritis was induced by intradermal injection of 200 μl of different hydrocarbon adjuvants at the base of the tail. Oil-induced arthritis was induced by intradermal injection of 300 μl Freund’s incomplete adjuvant. Collagen-induced arthritis was induced by intradermal injection of 100 µg collagen type II (CII) dissolved in 0.1 M acetic acid and emulsified in an equal volume of Freund’s incomplete adjuvant. Pristane (2,6,10,14-tetramethylpentadecane) were purchased from Acros Organics. Other hydrocarbon adjuvants pentadecane, phytol, hexadecane, hexadecane, heptadecane, squalane, squalene and arlacel A were purchased from Sigma Aldrich. Freund’s incomplete adjuvant was purchased from Difco, Detroit, MI, USA. Pepsin-digested CII from rat chondrosarcoma was prepared as previously described^52^. Arthritis development was monitored blindly using a macroscopic scoring system. One point is given for each inflamed knuckle or toe and up to five points were given for an affected ankle (maximum score per limb 15 and 60 for a rat). Weight change was used as an objective measurement of disease severity. Disease marker α-1-acid-glycoprotein (AGP) in serum diluted 1:20000 was measured by the AGP ELISA kit (Life Diagnostics). All experiments were approved and performed in accordance with the guidelines of the Swedish National Board for Laboratory Animals and the European Community Council Directive (86/609/EEC).

### Anti-TCR αβ treatment

The αβ T cell depleting mAb (R73) was started two days prior to ovalbumin administration at 200 μg, followed by 100 μg on days −1, 0, 2, 4, 6, 8, and 10. Control rats received an equal volume of PBS.

### Isolation of αβ CD4^+^ T cells

Lymph node single-cell suspensions was prepared in cold MACS buffer (0.5% BSA, 2 mM EDTA in PBS). NK cells, NK T cells, B cells, γδ T cells and CD8^+^ T cells in the cell suspension were labelled with mAb NK1.1 (10/78), CD45RA (OX33), CD8a (OX8) (all from BD Pharmingen) respectively and removed by magnetic depletion using Dynabeads Pan Mouse IgG. CD4^+^ T cells were then isolated using Pan T cell microbeads (Miltenyi Biotec) on MS-columns (Miltenyi Biotec). The purity of the isolated cells was generally > 98%.

### RNA extraction and quantitative real-time PCR (qRT-PCR)

RNA from CD4^+^ T cells was extracted by the RNeasy Mini kit (Qiagen, Ballerup, Denmark) and treated with DNase I (Roche, Mannhein, Germany). Complementary DNA was synthesized using High Capacity cDNA Reverse Transcription kit (ABI). QRT-PCR was performed on ABI 7900 HT (Applied Biosystems) using SYBR Green (Applied Biosystems) using a two-step PCR protocol (95 °C for 10 min, followed by 40 cycles for 95 °C for 10 sec and 60 °C for 30 sec). Primers were designed using NCBI/Primer-BLAST with sequences retrieved from public databases. Reference genes *Actb*, *Arbp* and *Hmbs* were used; and fold changes were determined using the standard comparative C_T_ method. Primer sequences are listed in Supplementary Table 1.

### Adoptive lymph node cell transfer

Lymph node cells from donor rats were harvested and cultured at a cell density 3 × 10^6^ per ml at 37 °C in DMEM medium (Gibco) supplemented with 5% FCS (Gibco), 50 μM 2-ME (Gibco), 10 mM HEPES (Gibco), 10U/ml penicillin and 100 μg/ml streptomycin (both Invitrogen Life Technologies) (‘complete medium’), together with 3 μg/ml Concanavalin A (Sigma Aldrich). After 65 hours, cells were collected, washed and resuspended in PBS; and 3 × 10^7^ cells were injected intravenously into each recipient rat.

### Ovalbumin immunisation and antibody ELISA

600 μg ovalbumin (from Sigma) dissolved in PBS and 200 μl adjuvant were injected to each rat at the base of the tail. Serum was taken from the rats 12 days after injection for antibody measurement. For serum antibody ELISA, the plate was coated with 10 μg/ml ovalbumin overnight at 4 °C in PBS. 50 μl serum at 1:250 to 1:10000 dilution was added to each well. Biotinylated anti-rat monoclonal antibodies against IgG2b (clone RG7/11.1; 2 μg/ml) (purchased from BD Pharmingen) were used as secondary reagents. Antibodies were detected using Eu^3+^-conjugated stretavidin (DELFIA) on a Synergy 2 multi-mode plate reader (BioTek).

### *In vitro* stimulation and cytokine ELISA

For the stimulation with plate-bound anti-CD3, 5 μg/ml anti-CD3 (1F4) was coated overnight at 4 °C in PBS. Lymph node cells were collected from rats on day 8 after injection, 5 × 10^5^ cells were added per well and cultured with 5 μg/ml anti-CD28 (JJ319) for 60 hours at 37 °C.

For cytokine detection, the level of IFN-γ in the culture supernatants was determined by ELISA using 2 μg/ml anti-rat IFN-γ (DB1, BioLegend) as capture antibodies; and biotinylated 0.5 μg/ml anti-rat IFN-γ (Poly5109, Biolegend) for detection. The level of IL-2 and IL-17 were determined using the Rat IL-2 Duoset ELISA Assay (DY502, R&D Systems) and the Mouse IL-17 DuoSet ELISA Assay (DY421, R&D Systems), respectively, according to the manufacturer’s instructions. Eu3^+^-conjugated stretavidin (DELFIA) was used for detection, performed on a Synergy 2 multi-mode plate reader (BioTek).

### Flow Cytometry

Lymph node single-cell suspensions in cold FACS buffer was stained with saturating concentration of MAbs and the following antibodies were used: CD8a (OX8), CD45RA (OX33), CD25 (OX39), CD71 (OX-26), ICAM-1 (1A29), RT1B (OX6), CD134 (OX40) were purchased from BD Pharmingen. CD3 (1F4) and CD4 (OX35), were purchased from Biolegend. αβTCR (R73), RT1D (OX17), CD27 (LG.7F9), Foxp3 (Fjk-16a), Ki67 (SolA15) and CD11b/c (OX42) were purchased from eBioscience. For the intracellular detection of Ki67 and Foxp3, cells were incubated for 45 minutes in Fixation/Permeabilization buffer (eBioscience) before washing in Permeabilization buffer (eBioscience) and staining with anti-Foxp3 and anti-Ki67. Both LIVE/Dead Violet (Invitrogen) and the forward scatter vs side scatter plot were used to include only non-necrotic cells. Acquisition was performed on a SORP BD LSR-II Analytic Flow Cytometer and data was analysed with Flowjo (Tree Star Inc., OR, USA).

### Generation of bead array

NeutrAvidin (Thermo Fischer) was coupled with carboxylated beads (COOH Microspheres, Luminex-Corp.) in accordance to previously published antigen coupling protocols with minor modifications^53–55^.

In brief, 10^6^ beads per bead identity were distributed across 96-well plates (Greiner BioOne), washed and re-suspended in phosphate buffer (0.1 M NaH_2_PO_4_, pH 6.2) using a plate magnet and a plate washer (EL406, Biotek). The carboxyl groups on the surface of the beads were activated by 0.5 mg of 1-ethyl-3(3-dimethylamino-propyl) carbodiimide (Pierce) and 0.5 mg of N-hydroxysuccinimide (Pierce) in 100 µl phosphate buffer. After 20 min incubation on a shaker (Grant Bio), beads were washed in MES buffer (0.05 M 2-(N-morpholino)ethanesulfonic acid, pH 5.0). 250 µg/ml of NeutraAvidin was prepared in MES buffer and added to the beads. The coupling reaction was allowed to take place for 2 h at room temperature (RT), the beads were then washed 3x in PBS-T (0.05% Tween20 in PBS), re-suspended in 100 µl storage buffer (1% BSA, 0.05% Tween20, ProClin300 in PBS) and stored in plates at 4 °C overnight. Following day, 100 µl of each different biotinylated peptide at 50 µM concentration was added to neutravidin-coated beads. Following an overnight incubation at 4 °C, beads were washed 3x in PBS-T and re-suspended in 100 µl storage buffer. The final antigen suspension bead array was prepared by combining equal volumes of each bead identity.

Immobilization of the peptides was confirmed by the use of monoclonal CII-epitope specific antibodies used in collagen antibody induced arthritis (data not shown).

### Assays on suspension bead arrays

Serum samples were diluted 1∶10 (v/v) in assay buffer (3% BSA, 5% milk powder, 0,1% ProClin300, 0,05% Tween 20, 100 μg/mL Neutravidin in PBS) and incubated for 60 min at RT on a shaker for pre-adsorption of unspecific antibodies.

Using a liquid handler (CyBi-SELMA, CyBio), 45 µl of 1∶10 diluted serum samples were transferred to a 384 well plate containing 5 µl bead array per well. Following incubation at RT on a shaker (Grant Bio) for 75 min, beads were washed 6x with 60 µl PBS-T on a plate washer (EL406, Biotek) and re-suspended in 50 µl of each secondary antibody solution. Anti-rat IgG Fcy–PE diluted 1∶500 in a buffer consisting of 5% BSA, 0.05% Tween20 in PBS and used for detection of antibodies. After incubation with the secondary antibodies for 30 min, the beads were washed 3x with 60 µl PBS-T and re-suspended in 60 µl PBS-T for measurement by a FlexMap3D instrument (Luminex Corp.). The median fluorescence intensity (MFI) was chosen to display serum antibody-peptide interactions. Arthritic serum samples were considered positive for a particular epitope if the MFI of the samples exceeded the mean MFI + 3 SD of the naïve rat samples.

### Bioinformatics and statistical analysis

Structure of oil adjuvants were obtained from https://pubchem.ncbi.nlm.nih.gov. For correlation between arthritis development and antibody response, a Spearman rank correlation coefficient, r, and the corresponding p value were calculated. Comparison of citrullinated versus non-citrullinated antibody response were determined with Wilcoxon matched-pairs signed rank test. All other statistical analysis was evaluated by Mann-Whitney *U* test and performed on Prism 6.0.

### Data availability statement

The datasets generated during and/or analysed during the current study are available from the corresponding author on reasonable request.

## Electronic supplementary material

Supplementary information
