# Supplementary material for: Influence of hydrocarbon oil structure on adjuvanticity and autoimmunity
Source: Sci Rep. 2017 Nov 8;7:14998. doi: 10.1038/s41598-017-15096-z (PMC5678145; doi:10.1038/s41598-017-15096-z)
Supplement: Supplementary file 1 — Supplementary information [file 41598_2017_15096_MOESM1_ESM.docx]

Title:

Influence of hydrocarbon oil structure on adjuvanticity and autoimmunity

Authors:

Anthony C. Y. Yau, Erik Lönnblom, Jianghong Zhong, Rikard Holmdahl

Supplementary Figure 1: Inguinal lymph nodes were harvested from rats 8 days after injection of pristane, hexadecane, squalane and squalene and stimulated with anti-CD3/CD28. After 60 hours, levels of cytokines (a) IFN-γ, (b) IL-17 were determined by ELISA (n = 6 - 7 per group). Data are shown as mean ± SEM. Statistics were determined with the Mann-Whitney *U* test. * denotes p < 0.05, ** denotes p < 0.01

Supplementary Figure 2: Immune cell frequency and expression of activation and proliferation markers on inguinal lymph nodes from DA rats primed with pristane, hexadecane, squalane and squalene for 8 days (n = 8 per group). (a) Total cell number, (b) B cells (%), (c) CD4^+^ T cells (%), (d) CD4^+^CD25^+^Foxp3^+^ T cells (%), (e) CD11b/c^int^ (%), (f) CD11b/c^high^ (%). (G-K) CD4^+^ T cell expression of (g) CD25^+^ (%), (h) CD27^+^ (MFI), (i) ICAM-1^+^(%, MFI), (j) CD71^+^ (%, MFI), (k) CD40^+^, (l) OX6^+^, (m) OX17, (n) Ki67^+^ (%) on CD4^+^ T cells and Ki67^+^ (%) on CD4^+^CD25^+^Foxp3^+^ T cells. Data are shown as mean ± SEM. Statistics were determined with the Mann-Whitney *U* test. * denotes p < 0.05, ** denotes p < 0.01, *** denotes p < 0.001.

Supplementary Figure 3: Antoantibody response in DA rats with CIA, non-citrullinated (R) versus citrullinated (Cit), against different epitopes (a) C1, (b) F4, (c) filaggrin, (d) α-enolase, (e) vimentin_1-20_, (f) vimentin_58-77_, (g) fibrinogen_60-78_, (h) fibrinogen_501-517_, (i) fibrinogen_617-635_. Statistics were determined with Wilcoxon matched-pairs signed rank test. * denotes p < 0.05, ** denotes p < 0.01, *** denotes p < 0.001.

Supplementary Figure 4: Antoantibody response in DA rats with PIA, non-citrullinated (R) versus citrullinated (Cit), against different epitopes (a) C1, (b) F4, (c) filaggrin, (d) α-enolase, (e) vimentin_1-20_, (f) vimentin_58-77_, (g) fibrinogen_60-78_, (h) fibrinogen_501-517_, (i) fibrinogen_617-635_. Statistics were determined with Wilcoxon matched-pairs signed rank test. * denotes p < 0.05, ** denotes p < 0.01, *** denotes p < 0.001.

Supplementary Figure 5: Molecular structure of phytol.

Supplementary Table 1 – Primers for quantitative real-time PCR

| Gene | Forward Primer | Reverse Primer |
| --- | --- | --- |
| *Actb* | GGGAAATCGTGCGTGACATT | GCGGCAGTGGCCATCTC |
| *Arbp* | GCTTCATTGTGGGAGCAGACA | CATGGTGTTCTTGCCCATCAG |
| *Hmbs* | TCTAGATGGCTCAGATAGCATGCA | TGGACCATCTTCTTGCTGAACA |
| *IFN-γ* | ATTCATGAGCATCGCCAAGTTC | TGACAGCTGGTGAATCACTCTGAT |
| *IL-17A* | CTCAGACTACCTCAACCGTTCC | GTGCCTCCCAGATCACAGAAG |
| *IL-21* | GGCTGCCTGCTAAGAGGACAGG | CACAGGAAGGGCATTTAGCCATGTG |
| *IL-22* | ATGCAGGAGGTGGTGCCTTTCC | TCACCGCTGATGTGACAGGGG |
| *IL4* | GCAACAAGGAACACCACGG | AAGCACGGAGGTACATCACGT |
| *IL-10* | TGCAACAGCTCAGCGCA | GTCACAGCTTTCGAGAGACTGGAA |
| *RT1Ba* | GACGACATTGAGGCCGACCAC | TCCAAACTCGGGGATCCTCCAG |
